# The Supply of Nurses Committee

**Published:** 1916-09-23

**Authors:** 


### THE MATRONS' AND SISTERS' DEPARTMENT.

### The Supply of Nurses Committee.

On September 15 the War Office issued to the Press an announcement which has already created no small degree of astonishment and a certain amount of indignation in nursing circles, especially amongst those who, as matrons, have administrative responsibilities. The statement in question intimated that a Committee has been appointed to consider the existing system of obtaining nurses for Army hospitals and to advise how the supply may be augmented. It also included the names of the seven members of the Committee, which has Mr. W. Bridgeman, M.P., as chairman.

The criticisms which have so far been voiced are directed against the secrecy and suddenness with which the Committee has been set up, against the vagueness of the duties it is expected to perform, against the necessity for any such Committee at all, and against the choice of its *personnel*. There is no evidence of the overwhelming urgency of the problem, so that there was no need for haste. It was therefore unwise of the War Office to adopt such a secretive policy, and was just the way to excite suspicion of ulterior motives and to engender a belief that severe criticism was both expected and unanswerable. We do not say that such was in fact the intention of whoever conceived and brought into being the Supply of Nurses Committee, for we know nothing about the matter; we merely remark that their methods are injudicious, and likely to defeat the purpose for which the Committee has been set up.

Further, it is being stated and appears to be widely believed that there has hitherto been no real difficulty in obtaining an adequate supply of nurses, fully trained, partly trained, and untrained, for the needs of the Army. There have been three principal channels of recruitment: the Queen Alexandra's Imperial Military Nursing Service, represented by its head, the Matron-in-Chief, at the War Office; the Territorial Force Nursing Service; and the Joint Committee of the British Red Cross and Order of St. John. The two former deal only with trained nurses, the latter, in addition, with two-year certificated nurses as staff nurses and V.A.D. members. Unless these sources of supply have dried up, or appear to be in danger of doing so, which is denied by many of those competent to judge, the necessity for a committee to supplement these authorities is not obvious. In the general bewilderment of the nursing profession when the announcement first appeared in the Press, it was suggested that possibly the Committee was being formed to deal with V.A.D. and untrained nurse problems alone; but according to the Secretary of the Committee, this is not the case. The vague language in which the reference is drawn leaves more room for speculation as to the exact purpose and functions of the Committee than is at all desirable in the public interest.

We now come to those criticisms which are the most numerous and, as influential members of the nursing profession think, the most serious: those, namely, that are directed against the constitution of the Committee. To deal first with omissions, where are the Matrons-in-Chief of the Regular and the Territorial Nursing Services, whose principal matrons have for two years been constantly and successfully engaged with the recruitment of trained nurses? Where is the Matron-in-Chief of the Joint Committee of the Red Cross and Order of St. John? Can it be seriously argued that Mrs. Furse, Commandant-in-Chief of the Women's V.A.D., and not a trained nurse, is to be regarded as specially charged with looking after that interest? Why is not a single matron or trained nurse of any sort placed upon a Committee whose object is mainly to obtain recruits from the nursing profession?

Can ineptitude go further it may well be asked. Apparently it can. Of the seven members of the Committee, four are directly connected with one particular nurse-training school (that of the London Hospital); and no other school in the entire Kingdom is represented at all! These four are Viscount Knutsford, Sir Frederick Treves, Captain Boulton, and Mr. E. W. Morris. No one questions Lord Knutsford's extraordinary knowledge of nursing matters, his ability or his honesty. He is also a member of the Nursing Board of the Q.A.I.M.N.S. But he is too deeply committed to views on certain nursing questions which are not generally accepted to be quite an ideal member of this particular Committee. Sir Frederick Treves has done much for the Joint Committee of the B.R.C.S. and the Order of St. John, but he represents the same training school as does Lord Knutsford. And the membership of Captain Boulton and Mr. Morris is surely superfluous; the former is one of the Committee of the London Hospital, the latter is the house governor there. It is true that Captain Boulton is vice-chairman of the Queen Victoria's Jubilee Institute for Nurses; but is that any reason for his inclusion in preference to some trained nurse or matron?

Altogether the whole situation is extraordinarily obscure. What is clear is that dissatisfaction with the action of the War Office is widespread—nay, universal—amongst the heads of the nursing profession. We cannot bring ourselves to credit that Mr. Lloyd George understood, when he signed the order, the objections to this Committee which have just been enumerated; and we feel sure that, when his attention is drawn to the points raised, he will make speedy amends. The plain fact is that he has slighted, not to say insulted, a very large body of professional women, from whom his department has met with nothing but loyalty and faithful service. That he had any such intention we do not for one moment believe, and we are convinced that, as soon as he realises what matrons and nurses feel about it, he will take effective measures to put himself right.

